# Hospital and Temporal Variations in Limitations of Care Among Hospitalized Patients with COVID-19: A VIRUS Registry Retrospective Cohort Study

**DOI:** 10.1177/26892820251380523

**Published:** 2025-09-25

**Authors:** Nathan Mesfin, Nitesh Kumar Jain, Anwar Khedr, Hisham Mushtaq, Abbas B. Jama, Noura Attallah, Esraa Hassan, Benjamin Langworthy, Nicholas Ingraham, Juan Pablo Domecq Garces, Thoyaja Koritala, Donna Lee Armaignac, Nicholas Eugene Perkins, Katherine Belden, Vishakha Kumar, Karen Boman, Devang K. Sanghavi, Vikas Bansal, Rahul Kashyap, Rodrigo Cartin-Ceba, Abigail La Nou, Allan J. Walkey, R. Adams Dudley, Syed Anjum Khan, on behalf of the Society of Critical Care Medicine Discovery Viral Infection and Respiratory Illness Universal Study (VIRUS): Covid-19 Registry Investigator Group

**Affiliations:** ^1^ Division of Pulmonary, Allergy, Critical Care, and Sleep Medicine, Department of Internal Medicine, University of Minnesota, Minneapolis, Minnesota, USA.; ^2^ Division of Critical Care Medicine, Department of Internal Medicine, Mayo Clinic Health System, Mankato, Minnesota, USA.; ^3^ Division of Biostatistics and Health Data Science, School of Public Health, University of Minnesota, Minneapolis, Minnesota, USA.; ^4^ Division of Nephrology and Hypertension, Department of Internal Medicine, Mayo Clinic, Rochester, Minnesota, USA.; ^5^ Department of Hospital Medicine, Mayo Clinic Health System, Mankato, Minnesota, USA.; ^6^ Center for Advanced Analytics, Baptist Health South Florida, Coral Gables, Florida, USA.; ^7^ Department of Hospital Medicine, Prisma Health, Greenville Memorial Hospital, Grenville, South Carolina, USA.; ^8^ Department of Medicine, Division of Infectious Disease, Thomas Jefferson University Sidney Kimmel Medical College, Philadelphia, Pennsylvania, USA.; ^9^ VIRUS Registry, Society of Critical Care Medicine, Mount Prospect, Illinois, USA.; ^10^ Department of Medicine, Mayo Clinic in Florida, Jacksonville, Florida, USA.; ^11^ Department of Anesthesia and Perioperative Medicine, Mayo Clinic, Rochester, Minnesota, USA.; ^12^ Department of Anesthesiology and Perioperative Medicine, Mayo Clinic, Rochester, Minnesota, USA.; ^13^ Division of Pulmonary Medicine, Department of Medicine, Mayo Clinic, Scottsdale, Arizona, USA.; ^14^ Department of Critical Care Medicine, Mayo Clinic Health System, Eau Claire, Wisconsin, USA.; ^15^ Pulmonary Center, Division of Pulmonary, Allergy, Critical Care and Sleep Medicine, Department of Medicine, Evans Center of Implementation and Improvement Sciences, Boston, University Chobanian and Avedisian School of Medicine, Boston, Massachusetts, USA.; ^16^ Institute of Health Informatics, University of Minnesota, Minneapolis, Minnesota, USA.

**Keywords:** code status, COVID-19, do-not-resuscitate, life support care, withholding treatment

## Abstract

**Introduction:**

Life-support limits (i.e., code status), the first step in the continuum of palliation and end-of-life (EoL) care, stem from decisions made by patients and their providers to align care near the EoL with patients’ wishes. The pandemic created health care strain that may have influenced life-support limitation practices among patients with serious COVID-19. In this study, we examine variations in life-support limitations (do-not-resuscitate, DNR; do-not-intubate, DNI) depending on the hospital and period of hospitalization.

**Methods:**

We included all adults admitted to a hospital in the United States with COVID-19 from January 2020 to December 2021 using the VIRUS registry. Our outcome was any life-support limitation on admission (DNR, DNI, or both) using regression modeling. Main exposures were hospital and period of hospitalization (early vs. late pandemic). Covariates included age, sex, race/ethnicity, comorbidities, and hospitalization diagnoses.

**Results:**

There were 42,383 patients from 75 hospitals in the US. The median age was 63 (interquartile range: 50–75), 46.5% were female, and 37.5% were non-Hispanic White. The life-support limitation rate was 7.9%, and the palliative care consultation rate was 2.3%. The odds of life-support limitation were 1.86 (95% CI: 1.6–2.1) depending on the hospital, but the period of hospitalization was not associated with odds of life-support limitation. Older age was associated with the greatest increased odds (4.1 per standard deviation ∼ 17.9 years, 95% CI: 3.9–4.4), followed by comorbidities including paralysis (2.4, 95% CI: 1.8–3.0) and dementia (2.3, 95% CI: 2.0–2.6).

**Conclusions:**

After adjusting for patient-level factors, we report significant inter-hospital but no temporal variation in life-support limitation upon admission with COVID-19. Future studies should investigate specific practices that led to health care resilience to temporal practice variations in life-support limitation during the pandemic.

Limiting care upon hospitalization (i.e., do not resuscitate [DNR], do not intubate [DNI], or [DNR/DNI]) is a decision between a patient and their provider that takes into consideration the patient’s preferences, values, comorbidities, and severity of illness.^1^ Limiting care during hospitalization represents one of the first steps in the continuum of palliative care near the end of life (EoL).^2^ This decision affects patients’ likelihood of survival and hospitals’ quality metrics for critical illness survival.^3^ A prior study demonstrated large variations in DNR rates between hospitals after accounting for patient characteristics.^4^ Additionally, hospitals with higher DNR use demonstrated lower health care utilization near the EoL.^3,4^ Moreover, palliative care services are crucial in bridging the gap between patients’ EoL care wishes and wish-concordant care near the EoL by improving communication. These conversations may culminate in limiting care during hospitalization, often captured by DNR or comfort-care orders and reduced health care utilization at the EoL.^5,6^ During the COVID-19 pandemic, dramatic increases in hospitalizations might have exacerbated hospital variation in DNR use, especially early in the pandemic.^7–9^ Some single-center studies observed increased DNR use during the pandemic compared to the pre-pandemic era.^7,10,11^ In this study we explored the influence of hospital of admission and period of admission on the decision to limit life support upon admission for COVID-19.

## Methods

We used the Society of Critical Care Medicine Discovery VIRUS registry of patients admitted to the hospital with COVID-19.^12–14^ We included all adults in the United States with confirmed SARS-CoV-2 infection. We excluded patients from non-US sites,^15^ patients without an admission code status, and hospitals with less than 30 patients in the registry. Our main exposures were hospital admission and period (“early” vs. “late”). “Early” were hospitalizations that occurred from January to September 2020 and “late” were those from October 2020 until December 2021.^16,17^ Our covariates included age, sex, race/ethnicity, comorbidities, and admission diagnoses.^18^ Life-support limitation was defined as the admission code status—any life-support limit (i.e., DNR, DNI, DNR/DNI, or comfort measures only designations) versus full care. For variables with greater than 10% missingness, we used multiple imputation by chained equations, with predictive mean matching for continuous variables and logistic regression for binary variables, excluding outcome and key exposure variables from imputation. A variance inflation factor was used to assess for multicollinearity with a cut-off value of 5. We developed a hierarchical logistic regression model with hospital-level random effects to determine the association between our exposure and life-support limitations.^19^ The temporal analysis used the same cohort but restricted it to those with a documented admission month/year, which we assigned to either the “early” or “late” period.

## Results

### Cohort characteristics

**FIG. 1.**
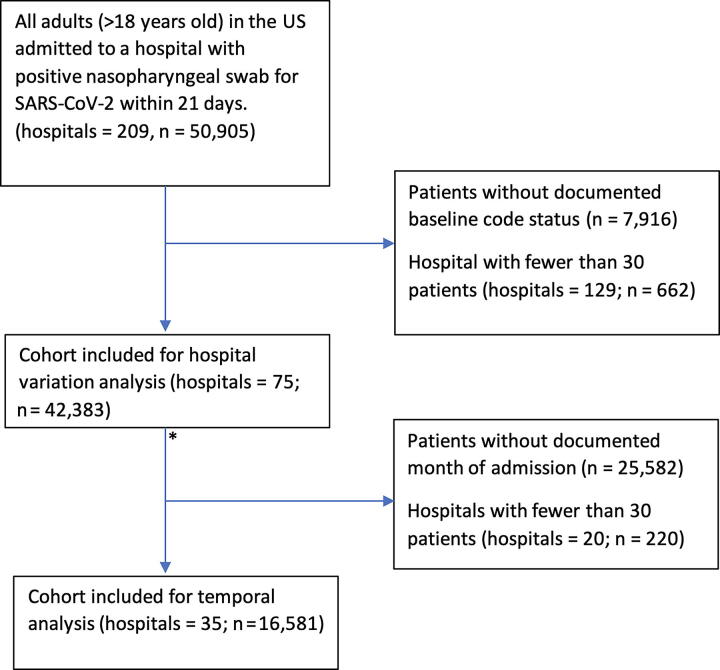
Flowchart for derivation of cohort for both hospital and temporal variation. The asterisk marks the point of missing data imputation.

There were 42,383 patients across 75 hospitals (Fig. 1). The median age was 63 (interquartile range [IQR] 50–75) years, 46.5% were female, 38.5% identified as Hispanic, and 30.1% were admitted to the intensive care unit (ICU) (see Table 1). The overall rate of life-support limitation on admission was 7.9%, and the rate of palliative care specialist involvement was 2.3%. We found that increased age (OR: 4.14, 95% CI: 3.89–4.42 per 1 SD ∼ 17.9 years) and female sex (OR: 1.32, 95% CI: 1.21–1.43) were associated with increased odds of life-support limitation. Asian (OR: 0.55, 95% CI: 0.41–0.75), Black or African American (OR: 0.52, 95% CI: 0.45–0.59), and Hispanic (OR: 0.65, 95% CI: 0.56–0.75) races had lower odds of life-support limitation compared to non-Hispanic White patients. Some comorbidities associated with increased odds of life-support limitation included paralysis (2.4, 95% CI: 1.8–3.0), dementia (OR: 2.30, 95% CI: 2.04–2.60), and metastatic cancer (OR: 1.59, 95% CI: 1.26–1.99).

**Table 1.** Baseline Characteristics and Demographics of Patients Admitted with COVID-19 by Baseline Code Status

	Total (*n* = 42,383)	Full code (*n* = 39,019)	Life-support limitation^c^ (*n* = 3364)
Age (median [IQR])	63 [50–75]	62 [49–73]	82 [73–89]
Sex (female)^a^	19,699 (46.5%)	17,861 (45.8%)	1838 (54.7%)
Race/Ethnicity			
non-Hispanic white	15881 (37.5%)	13851 (35.5%)	2030 (60.3%)
Black/AA	7470 (17.6%)	7049 (18.1%)	421 (12.5%)
Hispanic	16317 (38.5%)	15563 (39.9%)	754 (22.4%)
Asian	890 (2.1%)	829 (2.1%)	61 (1.8%)
Other	1237 (2.9%)	1172 (3%)	65 (1.9%)
Unknown	588 (1.4%)	555 (1.4%)	33 (1%)
Comorbidities^a^			
Chronic kidney disease	5816 (14.2%)	5065 (13.4%)	751 (23.2%)
Chronic pulmonary disease	6051 (14.8%)	5368 (14.2%)	683 (21.1%)
Congestive heart failure	4215 (10.3%)	3589 (9.5%)	626 (19.2%)
Coronary artery disease	4307 (10.5%)	3694 (9.8%)	613 (18.8%)
Diabetes mellitus	14232 (34.7%)	13052 (34.6%)	1180 (36.3%)
Hyperlipidemia	7525 (18.4%)	6730 (17.9%)	795 (24.6%)
Hypertension	23842 (58.1%)	21536 (57.0%)	2306 (70.9%)
Malignancy^b^	3102 (7.6%)	2816 (7.5%)	286 (8.9%)
Obesity	10078 (24.6%)	9716 (25.8%)	362 (11.1%)
Other	9031 (21.3%)	7976 (20.4%)	1055 (31.4%)
ICU hospitalization	11,176 (30.1%)	10,377 (30.2%)	799 (29.5%)
Hospitalization Diagnosis^a^			
Acute hypoxic respiratory failure	19414 (59.7%)	18153 (60.2%)	1261 (53.4%)
Acute renal failure without dialysis	2348 (7.2)	2068 (6.9)	280 (11.9)
Acute Respiratory Distress Syndrome	1077 (3.3%)	995 (3.3%)	82 (3.5%)
Atrial fibrillation	643 (2.0%)	536 (1.8%)	107 (4.5%)
Encephalopathy	868 (2.7%)	675 (2.2%)	193 (8.2%)
Hyperglycemia	863 (2.7%)	810 (2.7%)	53 (2.2%)
Other	15442 (47.5%)	14168 (47%)	1274 (53.9%)
Pneumonia	1380 (4.2%)	1232 (4.1%)	148 (6.3%)
Sepsis	2740 (8.4%)	2381 (7.9%)	359 (15.2%)
Shock	695 (2.1%)	626 (2.1%)	69 (2.9%)
In-hospital specialty services			
Palliative care services	988 (2.3%)	790 (2.0%)	198 (5.9%)

^a^ The 10 most common comorbidities and hospitalization diagnoses were included in this table.

^b^ Includes solid tumors without metastasis, hematological malignancies, and metastatic cancer.

^c^ Life-support limitation includes any limitation including do not resuscitate (DNR), do not intubate (DNI), DNR-DNI, or comfort care only.

Missing data were for age 7 (0.1%), sex 1 (0.0%), race/ethnicity 241 (0.5%), comorbidities 1362 (3%), ICU hospitalization 5278 (12%), and hospitalization diagnoses 9888 (23%).

IQR, interquartile range; ICU, intensive care unit.

### Hospital and temporal variation

**FIG. 2.**
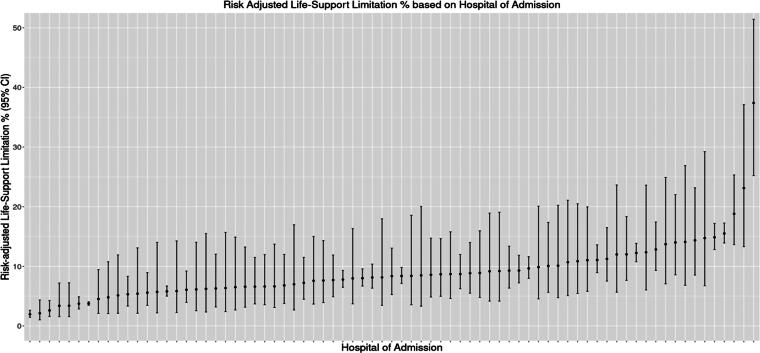
Hospitals ranked in order of increasing adjusted admission life support limitation rate (*n* = 42,383, 75 hospitals). The adjusted covariates include demographics, comorbidities, and admission diagnoses.

The median odds ratio for the effect of hospital of admission on life-support limitation was 1.86 (95% CI: 1.60–2.14) (FIG. 2 and Table 2). In the subset of patients for the temporal analysis, a total of 6653 (40.1%) were hospitalized during the early period and 9928 (59.9%) were hospitalized during the late period (see Supplemental Data). Those admitted during the late period were older, 65 (IQR: 53–76) versus 63 (IQR: 50–74). There were significantly more Black or African American and Hispanic patients admitted during the early period as compared to the late period (23.1% vs. 14.4% and 32.3% vs. 9.5%, respectively). In the adjusted multivariable model, there was no significant association between period of admission and life-support limitation (OR: 0.92, 95% CI: 0.79–1.07).

**Table 2.** Mixed-Effect Regression Model Results for Both Hospital and Temporal Variation

	OR (95% CI)	*p* value
Model 1 (*n* = 42,383)		
Hospital of admission^a^	1.85 (1.60–2.14)	<0.001
Model 2 (*n* = 16,801)		
Hospitalization during late pandemic (ref = early pandemic)	0.92 (0.79–1.07)	0.267

^a^ Refers to the median odds ratio.

Additional covariate results are reported in the Supplementary Material Appendices F and G.

## Discussion

Using a nationwide database, we report a large hospital variation on life-support limitation on admission for COVID-19 with a median odds ratio of 1.86 (CI: 1.60–2.14). In other words, the admitting hospital, on average, has as much influence on the odds of life-support limitation as does having metastatic malignancy. Walkey et al. previously demonstrated a nearly 2.65-fold difference in odds of a patient being DNR based on the hospital to which the person was admitted.^4^ These findings suggest that although life-support decisions are often driven by patients’ values for EoL care, hospital systems still significantly influence these decisions.

The influence of hospital systems on initial code status may reflect both pre- and early-hospitalization processes (i.e., outpatient advance care planning efforts and reliance on presumptive full code over nuanced discussions).^20,21^ A study previously demonstrated large regional variation in advance care planning visits, which could affect admission code status decisions.^22^ Furthermore, a systematic review found that patients with advance care plans are more likely to adopt comfort-oriented care (i.e., DNR status).^20^ A separate study found that in 53% of hospitalizations, clinicians did not address code status, resulting in continuation of the initial presumed full code throughout the entire hospitalization period.^21^ These suggest that health system processes can impact patients’ initial code status, which would invariably have cascading effects for health care utilization downstream. Yet despite the influence of health systems on a seemingly patient-centered decision, it was not necessarily perturbed by the COVID-19 pandemic as compared to the pre-pandemic era.^4^ It suggests hospitals have likely harmonized early processes for determining code status, allowing for some resilience to external pressures such as the COVID-19 pandemic. This study, nonetheless, highlights the need to identify processes of care early during hospitalizations that may lead to variations in inpatient code status (EHR trigger for high-risk patients to elicit code status discussions). Identifying processes of care that lead to lower health care utilization by identifying high-performing sites would be valuable.

As demonstrated in previous studies, we found Black or African American and Asian patients were less likely to have life-support limitation compared to non-Hispanic White patients, in keeping with prior literature.^23,24^ Interestingly, we found higher representation of Black/African American and Hispanic patients during the “early” as compared to the “late” period. This likely reflects the fact that historically minoritized groups are more likely to be essential workers and more likely to live in intergenerational homes, limiting their ability to work from home or quarantine during the early part of the pandemic.^25,26^

At the start of the pandemic, factors such as dramatic increases in hospitalizations and limited resources led to discussions around life-support limitation practices.^27–32^ Piscitello et al. found higher odds of DNR among patients admitted from April to June 2020 compared with those admitted July–September 2020.^10^ Comer et al. similarly found higher rates of DNR among patients admitted March–June 2020 as compared with those admitted July–October 2020.^11^ Epler et al. found that, of the patients admitted during the pandemic who had a pre-pandemic admission, nearly half elected some limitation of life support during the pandemic hospitalization.^7^ These studies suggest some deviation in practice patterns concerning life-support limits that may not have been present during the pre-pandemic or late pandemic time. In our multicenter, national study we found that the early pandemic period was not substantively different from the late period in terms of life-support limitation practices. We suspect that our use of a large, multicenter database likely reduced the likelihood of spurious associations that may arise from idiosyncrasies of single-center studies.

Additionally, our study found 2.3% of hospitalizations had palliative care specialty involvement. Kiker et al., using similar data, reported that 29% of decedents had palliative care consultation. Kodadek et al., using a multicenter cohort, reported around 25% of ICU patients received palliative care consultation.^33^ Our study’s lower rate of palliative care use likely reflects the inclusion of non-ICU patients and those with fewer comorbid conditions. Kiker et al. previously demonstrated slightly lower use of palliative care services during the pandemic as compared to pre-pandemic, highlighting an overburdened health care system.^5,24,34,35^

Although we found evidence of variation, this variation does not seem to have increased from pre-pandemic time.^4^ Additionally, despite waves of hospitalizations throughout the pandemic, admission life-support limitation practices likely did not change during the early pandemic. Future studies should examine specific practice patterns (early goals of care conversations) at individual sites that contributed to health care systems’ resilience to contextual pressures.

## Notes

**Cite this article as:** Mesfin N, Kumar Jain N, Mushtaq H, Jama AB, Attallah N, Hassan E, Langworthy B, Ingraham N, Garces JP D, Koritala T, Armaignac DL, Perkins NE, Belden K, Kumar V, Boman K, Sanghavi DK, Bansal V, Kashyap R, Cartin-Ceba R, Nou AL, Walkey AJ, Dudley RA (2025) Hospital and temporal variations in limitations of care among hospitalized patients with COVID-19: A virus registry retrospective cohort study, *Palliative Medicine Reports* 6:1, 468–473, DOI: 10.1177/26892820251380523.

## Abbreviations Used

DNI: do not intubate
DNR: Do not resuscitate
EoL: End-of-life
