# Supplementary material for: Hospital and Temporal Variations in Limitations of Care Among Hospitalized Patients with COVID-19: A VIRUS Registry Retrospective Cohort Study
Source: Palliat Med Rep. 2025 Sep 25;6(1):468–73. doi: 10.1177/26892820251380523 (PMC12547400; doi:10.1177/26892820251380523)
Supplement: Supplementary Data [file 26892820251380523_suppl_data.docx]

**Supplemental Material:**

[**Appendix A**](#A) **–** List of comorbidities and admission diagnoses used to assess inter-hospital and temporal variation in life support limitation.

[**Appendix B**](#B) **–** Table 1, race/ethnicity cross tabulation and algorithm used to group race/ethnicity categories for final analysis.

[**Appendix C**](#C) **–** Table 2, baseline characteristics and demographics of patients admitted with Covid-19 illness by baseline code status.

**[Appendix D](#D) –** Figure 1, monthly admission counts across pandemic months.

[**Appendix E**](#E) **–** Table 3, baseline characteristics and demographics of patients admitted with Covid-19 illness stratified by early versus late pandemic admission.

[**Appendix F**](#F) **–** Table 4, Mixed-effect regression model odds ratio for exposure variable and all covariates for hospital variation.

[**Appendix G**](#G) **–** Table 5, Mixed-effect regression model odds ratio for exposure variable and all covariates for temporal variation.

[**Appendix H**](#H) **–** Table 6, Mixed-effect regression model odds ratio for exposure variable for both models.

[**Appendix I**](#I) **–** Figure 2, all covariates odds ratio for likelihood of having life support limitation on admission (n = 42,3838). Figure 3, the unadjusted and adjusted monthly rate of life-support limitation amongst cohort with admission information available (n = 16,581)

[**Appendix J**](#J) **–** Figure 4, receiver operating curve for predicting life support limitation upon admission based on only baseline demographic, then with added comorbidities and admission diagnoses, and then with addition of hospital of admission. Figure 5, calibration plot for final model which includes baseline characteristics, comorbidities, diagnoses, and hospital of admission.

[**Appendix K**](#K) **–** Full list of all contributing collaborators to the VIRUS registry that enabled this manuscript.

**Appendix A**

**VIRUS Registry (source)** - A prospective, cross-sectional, multi-center international observational studies of patients admitted to the hospital for Covid-19 illness.

| 1, None \| 2, Coronary artery disease \| 3, Hypertension \| 4, Cardiac arrhythmias \| 5, Congestive heart failure \| 6, Valvular heart disease \| 7, Chronic pulmonary disease (not asthma) \| 8, Asthma (physician diagnosed) \| 9, Pulmonary circulation disorder \| 10, Chronic Kidney Disease \| 11, Chronic dialysis \| 12, Diabetes \| 13, Hypothyroidism \| 14, Liver disease \| 15, Hepatitis B \| 16, Hepatitis C \| 17, Peptic ulcer disease excluding bleeding \| 18, Solid tumor without metastasis \| 19, Hematologic malignancy \| 20, Metastatic cancer \| 21, History of solid organ or bone marrow transplant \| 22, HIV/AIDS or other immunosuppression \| 23, Stroke or other neurological disorders \| 24, Paralysis \| 25, Rheumatoid arthritis/collagen vascular disease \| 26, Blood loss anemia \| 27, Iron deficiency anemia \| 28, Coagulopathy \| 29, Malnutrition \| 30, Obesity \| 31, Substance use disorder \| 32, Depression \| 33, Psychosis \| 34, Dementia \| 35, Obstructive Sleep Apnea on Home CPAP/Bi-PAP use \| 36, Venous thromboembolism \| 51, Other |
| --- |

These comorbidities were used in the inter-hospital life-support limitation and temporal variation analysis.

| 1, Acute Hypoxic Respiratory Failure (Non-ARDS) \| 2, Acute Liver Injury \| 3, Acute Myocardial Infarction \| 4, Acute Renal Failure Requiring Hemofiltration \| 5, Acute Renal Injury, No Hemofiltration \| 6, Acute Respiratory Distress Syndrome \| 7, Bacteremia \| 8, Bacterial Pneumonia \| 9, Cardiac Arrest \| 10, Cardiac Arrhythmia: Atrial Fibrillation \| 11, Cardiac Arrhythmia: Heart Block \| 12, Cardiac Arrhythmia: Torsades Des Point \| 13, Cardiac Arrhythmia: Ventricular Tachycardia \| 14, Congestive Heart Failure / Cardiomyopathy \| 15, Delirium / Encephalopathy \| 16, Disseminated Intravascular Coagulation \| 17, Gastrointestinal Hemorrhage \| 18, Hyperglycemia \| 19, Hypoglycemia \| 20, Meningitis/Encephalitis \| 21, Myocarditis \| 22, Pneumothorax \| 23, Pleural Effusion \| 24, Rhabdomyolysis / Myositis \| 25, Seizure \| 26, Sepsis \| 27, Shock \| 28, Stroke \| 29, Diabetic Ketoacidosis (DKA) \| 30, Other |
| --- |

These admission diagnoses were used in the inter-hospital life-support limitation and temporal variation analysis.

**Appendix B**

**Table 1:** Race and ethnicity cross tabulation and algorithm utilized to reduce variables.

| **Ethnicity** | 1. Unknown | 1. Hispanic | 1. Non-Hispanic | 1. Not applicable |
| --- | --- | --- | --- | --- |
| **Race** |  |  |  |  |
| 1. American Indian or Alaska Native | 3 | 20 | 273 | 2 |
| 1. Asian American | 8 | 6 | 608 | 2 |
| 1. Black or African American | 145 | 410 | 7230 | 75 |
| 1. Native Hawaiian or Other Pacific Islander | 3 | 20 | 45 | 0 |
| 1. White Caucasian | 353 | 14159 | 15363 | 58 |
| 1. Other | 260 | 1212 | 485 | 11 |
| 1. East Asian (China, Hongkong, Japan, S. Korea, etc.) | 1 | 1 | 63 | 2 |
| 1. South Asian (India, Pakistan, Sri Lanka etc.) | 2 | 0 | 64 | 3 |
| 1. West Asian (including Arabic) | 0 | 0 | 13 | 0 |
| 1. South East Asian (Philippines, Thailand, Malaysia, Singapore, Vietnam etc.) | 0 | 0 | 120 | 2 |
| 1. Mixed Race | 45 | 364 | 110 | 0 |
| 1. Unknown | 213 | 124 | 166 | 3 |

Code utilized to reduce field:

mycohort$race_ethnicity<-ifelse(mycohort$race == 5 & (mycohort$ethnicity == 0 | mycohort$ethnicity == 2 | mycohort$ethnicity == 3 | is.na(mycohort$ethnicity)),"white",ifelse(mycohort$race == 3 & (mycohort$ethnicity == 0 | mycohort$ethnicity == 2 | mycohort$ethnicity == 3 | is.na(mycohort$ethnicity)),"Black or African American",ifelse((mycohort$race == 2 | mycohort$race == 7 | mycohort$race == 8 | mycohort$race == 9 | mycohort$race == 10) & (mycohort$ethnicity == 0 | mycohort$ethnicity == 2 | mycohort$ethnicity == 3| is.na(mycohort$ethnicity)),"Asian",ifelse(mycohort$ethnicity == 1,"Hispanic",ifelse((mycohort$race == 1 | mycohort$race == 4 | mycohort$race == 6 | mycohort$race == 11) & (mycohort$ethnicity == 0 | mycohort$ethnicity == 2 | mycohort$ethnicity == 3 | is.na(mycohort$ethnicity)),"Other",ifelse((mycohort$race == 12 | is.na(mycohort$race)) & (mycohort$ethnicity == 0 | mycohort$ethnicity == 2 | mycohort$ethnicity == 3 | is.na(mycohort$ethnicity)),"Unknown",NA))))))

**Appendix C**

| Table 2: Baseline characteristics and demographics of patients admitted with Covid-19 illness by baseline code status. | | | | |
| --- | --- | --- | --- | --- |
|  | Total  (n = 42,383) | Full Code  (n = 39,019) | Life support limitation*  (n = 3,364) | *p*-value |
| age (median [IQR]) | 63 [50-75] | 62 [49-73] | 82 [73-89] | <0.001 |
| Sex (female)*^†^* | 19,699 (46.5%) | 17,861 (45.8%) | 1838 (54.7%) | <0.001 |
| Race/ethnicity |  |  |  |  |
| *non-Hispanic white* | 15881 (37.5%) | 13851 (35.5%) | 2030 (60.3%) | <0.001 |
| *Black / AA* | 7470 (17.6%) | 7049 (18.1%) | 421 (12.5%) |  |
| *Hispanic* | 16317 (38.5%) | 15563 (39.9%) | 754 (22.4%) |  |
| *Asian* | 890 (2.1%) | 829 (2.1%) | 61 (1.8%) |  |
| *Other* | 1237 (2.9%) | 1172 (3%) | 65 (1.9%) |  |
| *Unknown* | 588 (1.4%) | 555 (1.4%) | 33 (1%) |  |
| Comorbidities* |  |  |  |  |
| *Arrhythmia* | 2942 (7.2%) | 2510 (6.6%) | 432 (13.3%) | <0.001 |
| *Asthma* | 3163 (7.7%) | 2966 (7.9%) | 197 (6.1%) | <0.001 |
| *Blood loss anemia* | 629 (1.5%) | 561 (1.5%) | 68 (2.1%) | 0.009 |
| *Chronic kidney disease* | 5816 (14.2%) | 5065 (13.4%) | 751 (23.1%) | <0.001 |
| *Chronic pulmonary disease* | 6051 (14.8%) | 5368 (14.2%) | 683 (21.0%) | <0.001 |
| *Coagulopathy* | 1790 (4.4%) | 1643 (4.4%) | 147 (4.5%) | 0.684 |
| *Congestive heart failure* | 4215 (10.3%) | 3589 (9.5%) | 626 (19.2%) | <0.001 |
| *Coronary artery disease* | 4307 (10.5%) | 3694 (9.8%) | 613 (18.8%) | <0.001 |
| *Diabetes mellitus* | 14232 (34.7%) | 13052 (34.6%) | 1180 (36.3%) | 0.051 |
| *Dementia* | 2266 (5.5%) | 1551 (4.1%) | 715 (22.0%) | <0.001 |
| *Depression* | 4131 (10.1%) | 3729 (9.9%) | 402 (12.4%) | <0.001 |
| *End-stage renal disease* | 1143 (2.8%) | 1052 (2.8%) | 91 (2.8%) | 1.000 |
| *Hematologic malignancy* | 1599 (3.9%) | 1530 (4.1%) | 69 (2.1%) | <0.001 |
| *Hepatitis B* | 112 (0.3%) | 99 (0.3%) | 13 (0.4%) | 0.205 |
| *Hepatitis C* | 517 (1.3%) | 464 (1.2%) | 53 (1.6%) | 0.060 |
| *HIV/AIDS or other immunosuppression* | 249 (0.6%) | 233 (0.6%) | 16 (0.5%) | 0.445 |
| *Hyperlipidemia* | 7525 (18.3%) | 6730 (17.8%) | 795 (24.4%) | <0.001 |
| *Hypertension* | 23842 (58.1%) | 21536 (57.0%) | 2306 (70.9%) | <0.001 |
| *Hypothyroidism* | 3954 (9.6%) | 3435 (9.1%) | 519 (16.0%) | <0.001 |
| *Iron deficiency anemia* | 4169 (10.2%) | 3709 (9.8%) | 460 (14.1%) | <0.001 |
| *Liver disease* | 2398 (5.8%) | 2289 (6.1%) | 109 (3.4%) | <0.001 |
| *Metastatic cancer* | 777 (1.9%) | 661 (1.8%) | 116 (3.6%) | <0.001 |
| *Malnutrition* | 501 (1.2%) | 401 (1.0%) | 100 (3.1%) | <0.001 |
| *Obesity* | 10078 (24.6%) | 9716 (25.7%) | 362 (11.1%) | <0.001 |
| *Obstructive sleep apnea or obesity hypoventilation syndrome* | 2227 (5.4%) | 2054 (5.4%) | 173 (5.3%) | 0.802 |
| *Organ or bone marrow transplant* | 306 (0.7%) | 290 (0.8%) | 16 (0.5%) | 0.099 |
| *Other* | 9031 (21.2%) | 7976 (21.1%) | 1055 (32.4%) | <0.001 |
| *Paralysis* | 589 (1.4%) | 467 (1.2%) | 122 (3.8%) | <0.001 |
| *Peptic ulcer disease* | 251 (0.6%) | 213 (0.6%) | 38 (1.2%) | <0.001 |
| *Pulmonary circulation disorder* | 648 (1.6%) | 606 (1.6%) | 42 (1.3%) | 0.193 |
| *Psychosis* | 1137 (2.8%) | 1073 (2.8%) | 64 (2.0%) | 0.004 |
| *Rheumatologic or collagen vascular disorder* | 827 (2.0%) | 741 (2.0%) | 86 (2.6%) | 0.010 |
| *Solid tumors without metastasis* | 1395 (3.4%) | 1244 (3.3%) | 151 (4.6%) | <0.001 |
| *Stroke or other neurological disorders* | 3568 (8.7%) | 3027 (8.0%) | 541 (16.6%) | <0.001 |
| *Substance use disorder* | 1685 (4.1%) | 1603 (4.2%) | 82 (2.5%) | <0.001 |
| *Valvulopathy* | 1666 (4.1%) | 1453 (3.8%) | 213 (6.5%) | <0.001 |
| *Venous thromboembolism* | 1417 (3.5%) | 1260 (3.3%) | 157 (4.8%) | <0.001 |
| ICU admission | 11,176 (30.1%) | 10,377 (30.2%) | 799 (29.5%) | 0.482 |
| Admission Diagnosis* |  |  |  |  |
| *Acute hypoxic respiratory failure* | 19414 (59.7%) | 18153 (60.2%) | 1261 (53.4%) | <0.001 |
| *Acute liver injury* | 256 (0.8%) | 239 (0.8%) | 17 (0.7%) | 0.789 |
| *Acute myocardial injury* | 202 (0.6%) | 178 (0.6%) | 24 (1%) | 0.017 |
| *Acute renal failure without dialysis* | 2348 (7.2) | 2068 (6.9) | 280 (11.9) | <0.001 |
| *Acute renal failure with dialysis* | 283 (0.9%) | 252 (0.8%) | 31 (1.3%) | 0.022 |
| *Acute Respiratory Distress Syndrome* | 1077 (3.3%) | 995 (3.3%) | 82 (3.5%) | 0.701 |
| *Atrial fibrillation* | 643 (2.0%) | 536 (1.8%) | 107 (4.5%) | <0.001 |
| *Bacteremia* | 169 (0.5%) | 154 (0.5%) | 15 (0.6%) | 0.510 |
| *Cardiac arrest* | 154 (0.5%) | 142 (0.5%) | 12 (0.5%) | 0.924 |
| *Congestive heart failure* | 526 (1.6%) | 450 (1.5%) | 76 (3.2%) | <0.001 |
| *Diabetic ketoacidosis* | 109 (0.3%) | 101 (0.3%) | 8 (0.3%) | 1.000 |
| *Disseminated intravascular coagulation* | 13 (0.0%) | 13 (0.0%) | 0 (0.0%) | 0.634 |
| *Encephalopathy* | 868 (2.7%) | 675 (2.2%) | 193 (8.2%) | <0.001 |
| *Gastrointestinal bleeding* | 198 (0.6%) | 185 (0.6%) | 13 (0.6%) | 0.806 |
| *Heart block* | 66 (0.2%) | 62 (0.2%) | 4 (0.2%) | 0.888 |
| *Hyperglycemia* | 863 (2.7%) | 810 (2.7%) | 53 (2.2%) | 0.220 |
| *Hypoglycemia* | 111 (0.3%) | 94 (0.3%) | 17 (0.7%) | 0.002 |
| *Meningoencephalitis* | 17 (0.1%) | 15 (0.0%) | 2 (0.1%) | 0.805 |
| *Myocarditis* | 20 (0.1%) | 18 (0.1%) | 2 (0.1%) | 0.968 |
| *Other* | 15442 (47.5%) | 14168 (47%) | 1274 (53.9%) | <0.001 |
| *Pleural effusion* | 197 (0.6%) | 172 (0.6%) | 25 (1.1%) | 0.005 |
| *Pneumonia* | 1380 (4.2%) | 1232 (4.1%) | 148 (6.3%) | <0.001 |
| *Pneumothorax* | 59 (0.2%) | 54 (0.2%) | 5 (0.2%) | 0.915 |
| *Rhabdomyolysis* | 87 (0.3%) | 76 (0.3%) | 11 (0.5%) | 0.084 |
| *Seizure* | 119 (0.4%) | 110 (0.4%) | 9 (0.4%) | 1.000 |
| *Sepsis* | 2740 (8.4%) | 2381 (7.9%) | 359 (15.2%) | <0.001 |
| *Shock* | 695 (2.1%) | 626 (2.1%) | 69 (2.9%) | 0.008 |
| *Stroke* | 223 (0.7%) | 201 (0.7%) | 22 (0.9%) | 0.171 |
| *Torsades des point* | 66 (0.2%) | 61 (0.2%) | 5 (0.2%) | 1.000 |
| *Ventricular tachycardia* | 141 (0.4%) | 127 (0.4%) | 14 (0.6%) | 0.291 |
| ICU, intensive care unit  *Life support limitation includes any limitation including do not resuscitate (DNR), do not intubate (DNI), DNR-DNI, or comfort care only.  Missing data were for age 7 (0.1%), sex 1 (0.0%), race/ethnicity 241 (0.5%), comorbidities 1362 (3%), ICU admission 5278 (12%), and admission diagnoses 9888 (23%) | | | | |

**Appendix D**

|  | Early Pandemic | | | | | | | | | Late Pandemic | | | | | | | | | | | | | | |
| --- | --- | --- | --- | --- | --- | --- | --- | --- | --- | --- | --- | --- | --- | --- | --- | --- | --- | --- | --- | --- | --- | --- | --- | --- |
| Month of admission | January 2020 | February 2020 | March 2020 | April 2020 | May 2020 | June 2020 | July 2020 | August 2020 | September 2020 | October 2020 | November 2020 | December 2020 | January 2021 | February 2021 | March 2021 | April 2021 | May 2021 | June 2021 | July 2021 | August 2021 | September 2021 | October 2021 | November 2021 | December 2021 |
| Admission count | 29 | 5 | 415 | 1123 | 880 | 915 | 1936 | 838 | 700 | 1233 | 2109 | 2193 | 1384 | 480 | 291 | 377 | 256 | 66 | 44 | 198 | 317 | 362 | 372 | 278 |

**Figure 1:** Monthly admission counts across the entire pandemic months for the VIRUS registry. Black bar represents cut-off point for early and late pandemic.

**
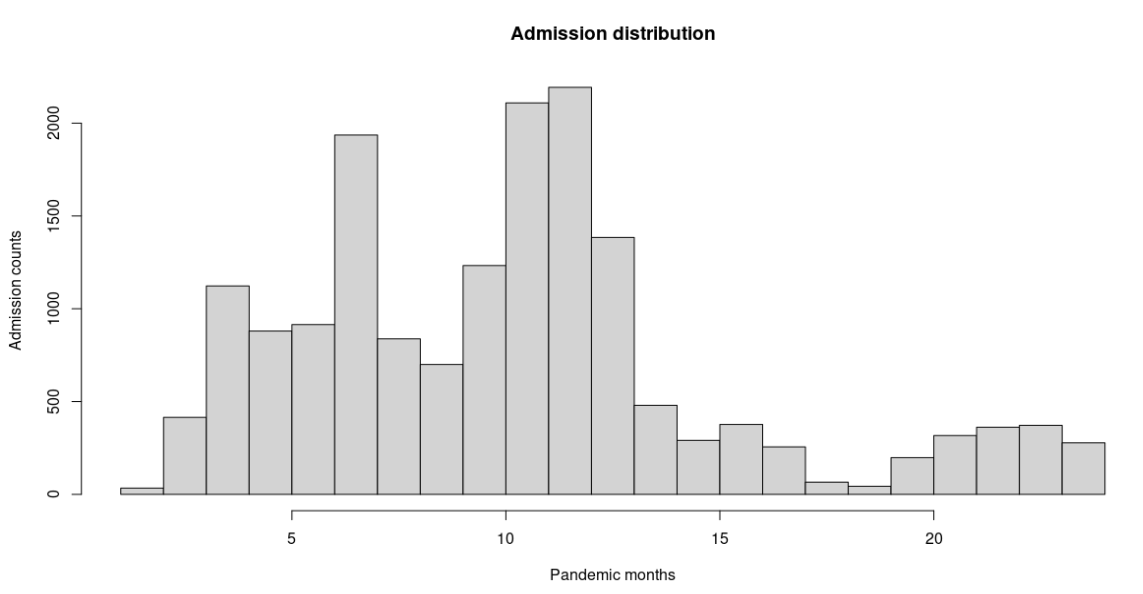
**

**Appendix E**

| Table 3: Baseline characteristics and demographics of patients admitted with Covid-19 illness stratified by early versus late pandemic admission. | | | | |
| --- | --- | --- | --- | --- |
|  | Total  (n = 16,581) | Early Pandemic  (n = 6,653) | Late Pandemic  (n = 9,928) | *p*-value |
| age (median [IQR]) | 64 [52-76] | 63 [50-74] | 65 [53-76] | <0.001 |
| Sex (female)*^†^* | 7544 (45.5%) | 3110 (46.7%) | 4434 (44.7%) | 0.009 |
| Race/ethnicity |  |  |  |  |
| *non-Hispanic white* | 9058 (54.6%) | 2445 (36.8%) | 3349(33.7%) | <0.001 |
| *Black / AA* | 2966 (17.9 %) | 1540 (23.1%) | 1426 (14.4%) |  |
| *Hispanic* | 3090 (18.6%) | 2148 (32.3%) | 942 (9.5%) |  |
| *Asian* | 453 (2.7%) | 184 (2.8%) | 269 (2.7%) |  |
| *Other* | 463 (2.8%) | 232 (3.5%) | 231 (2.3%) |  |
| *Unknown* | 551 (3.3%) | 163 (2.5%) | 388 (3.9%) |  |
| Comorbidities* |  |  |  |  |
| *Arrhythmia* | 1127 (6.8%) | 499 (7.5%) | 628 (6.3%) | 0.004 |
| *Asthma* | 1948 (11.7%) | 728 (10.9%) | 1220 (12.3%) | 0.009 |
| *Blood loss anemia* | 196 (1.2%) | 68 (1.0%) | 128 (1.3%) | 0.137 |
| *Chronic kidney disease* | 2671 (16.1%) | 1025 (15.4%) | 1646 (16.6%) | 0.046 |
| *Chronic pulmonary disease* | 2326 (14.0%) | 928 (13.9%) | 1398 (14.1%) | 0.827 |
| *Coagulopathy* | 476 (2.9%) | 173 (2.6%) | 303 (3.1%) | 0.097 |
| *Congestive heart failure* | 1559 (9.4%) | 588 (8.8%) | 971 (9.8%) | 0.044 |
| *Coronary artery disease* | 2401 (14.5%) | 864 (13%) | 1537 (15.5%) | <0.001 |
| *Diabetes mellitus* | 5737 34.6%) | 2362 (35.5%) | 3375 (34%) | 0.047 |
| *Dementia* | 752 (4.5%) | 434 (6.5%) | 318 (3.2%) | <0.001 |
| *Depression* | 1987 (12.0%) | 721 (10.8%) | 1266 (12.8%) | <0.001 |
| *End-stage renal disease* | 469 (2.8%) | 209 (3.1%) | 260 (2.6%) | 0.052 |
| *Hematologic malignancy* | 937 (5.7%) | 288 (4.3%) | 649 (6.5%) | <0.001 |
| *Hepatitis B* | 57 (0.3%) | 25 (0.4%) | 32 (0.3%) | 0.659 |
| *Hepatitis C* | 186 (1.1%) | 89 (1.3%) | 97 (1.0%) | 0.037 |
| *HIV/AIDS or other immunosuppression* | 136 (0.8%) | 71 (1.1%) | 65 (0.7%) | 0.005 |
| *Hyperlipidemia* | 5252 (31.7%) | 1737 (26.1%) | 3515 (35.4%) | <0.001 |
| *Hypertension* | 9546 (57.6%) | 3851 (57.9%) | 5695 (57.4%) | 0.516 |
| *Hypothyroidism* | 1294 (7.8%) | 549 (8.3%) | 745 (7.5%) | 0.084 |
| *Iron deficiency anemia* | 927 (5.6%) | 437 (6.6%) | 490 (4.9%) | <0.001 |
| *Liver disease* | 616 (3.7%) | 242 (3.6%) | 374 (3.8%) | 0.696 |
| *Metastatic cancer* | 442 (2.7%) | 112 (1.7%) | 330 (3.3%) | <0.001 |
| *Malnutrition* | 156 (0.9%) | 74 (1.1%) | 82 (0.8%) | 0.073 |
| *Obesity* | 3192 (19.3%) | 1367 (20.5%) | 1825 (18.4%) | 0.001 |
| *Obstructive sleep apnea or obesity hypoventilation syndrome* | 1491 (9.0%) | 544 (8.2%) | 947 (9.5%) | 0.003 |
| *Organ or bone marrow transplant* | 211 (1.3%) | 69 (1.0%) | 142 (1.4%) | 0.032 |
| *Other* | 6802 (41.0%) | 2924 (44.0%) | 3878 (39.1%) | <0.001 |
| *Paralysis* | 85 (0.5%) | 45 (0.7%) | 40 (0.4%) | 0.021 |
| *Peptic ulcer disease* | 105 (0.6%) | 54 (0.8%) | 51 (0.5%) | 0.023 |
| *Pulmonary circulation disorder* | 113 (0.7%) | 49 (0.7%) | 64 (0.6%) | 0.543 |
| *Psychosis* | 635 (3.8%) | 239 (3.6%) | 396 (4.0%) | 0.207 |
| *Rheumatologic or collagen vascular disorder* | 340 (2.1%) | 125 (1.9%) | 215 (2.2%) | 0.222 |
| *Solid tumors without metastasis* | 574 (3.5%) | 212 (3.2%) | 362 (3.6%) | 0.123 |
| *Stroke or other neurological disorders* | 1267 (7.6%) | 578 (8.7%) | 689 (6.9%) | <0.001 |
| *Substance use disorder* | 660 (4.0%) | 214 (3.2%) | 446 (4.5%) | <0.001 |
| *Valvulopathy* | 441 (2.7%) | 154 (2.3%) | 287 (2.9%) | 0.027 |
| *Venous thromboembolism* | 741 (4.5%) | 291 (4.4%) | 450 (4.5%) | 0.655 |
| ICU admission | 5794 (34.9%) | 2445 (36.8%) | 3349 (33.7%) | <0.001 |
| Admission Diagnosis |  |  |  |  |
| *Acute hypoxic respiratory failure* | 7863 (47.4%) | 3532 (53.1%) | 4331 (43.6%) | <0.001 |
| *Acute liver injury* | 198 (1.2%) | 89 (1.3%) | 109 (1.1%) | 0.187 |
| *Acute myocardial injury* | 249 (1.5%) | 60 (0.9%) | 189 (1.9%) | <0.001 |
| *Acute renal failure without dialysis* | 2004 (12.1%) | 872 (13.1%) | 1132 (11.4%) | 0.001 |
| *Acute renal failure with dialysis* | 357 (2.2%) | 146 (2.2%) | 211 (2.1%) | 0.805 |
| *Acute Respiratory Distress Syndrome* | 811 (4.9%) | 359 (5.4%) | 452 (4.6%) | 0.015 |
| *Atrial fibrillation* | 690 (4.2%) | 262 (3.9%) | 428 (4.3%) | 0.255 |
| *Bacteremia* | 243 (1.5%) | 99 (1.5%) | 144 (1.5%) | 0.895 |
| *Cardiac arrest* | 300 (1.8%) | 89 (1.3%) | 211 (2.1%) | <0.001 |
| *Congestive heart failure* | 530 (3.2%) | 190 (2.9%) | 340 (3.4%) | 0.046 |
| *Diabetic ketoacidosis* | 158 (1.0%) | 58 (0.9%) | 100 (1.0%) | 0.425 |
| *Disseminated intravascular coagulation* | 155 (0.9%) | 29 (0.4%) | 126 (1.3%) | <0.001 |
| *Encephalopathy* | 821 (5.0%) | 343 (5.2%) | 478 (4.8%) | 0.339 |
| *Gastrointestinal bleeding* | 249 (1.5%) | 71 (1.1%) | 178 (1.8%) | <0.001 |
| *Heart block* | 207 (1.2%) | 49 (0.7%) | 158 (1.6%) | <0.001 |
| *Hyperglycemia* | 845 (5.1%) | 368 (5.5%) | 477 (4.8%) | 0.040 |
| *Hypoglycemia* | 257 (1.5%) | 74 (1.1%) | 183 (1.8%) | <0.001 |
| *Meningoencephalitis* | 205 (1.2%) | 45 (0.7%) | 160 (1.6%) | <0.001 |
| *Myocarditis* | 221 (1.3%) | 44 (0.7%) | 177 (1.8%) | <0.001 |
| *Other* | 12386 (74.7%) | 4914 (73.9%) | 7472 (75.3%) | 0.044 |
| *Pleural effusion* | 398 (2.4%) | 121 (1.8%) | 277 (2.8%) | <0.001 |
| *Pneumonia* | 1265 (7.6%) | 471 (7.1%) | 794 (8.0%) | 0.031 |
| *Pneumothorax* | 271 (1.6%) | 77 (1.2%) | 194 (2.0%) | <0.001 |
| *Rhabdomyolysis* | 92 (0.6%) | 47 (0.7%) | 45 (0.5%) | 0.041 |
| *Seizure* | 151 (0.9%) | 58 (0.9%) | 93 (0.9%) | 0.728 |
| *Sepsis* | 1844 (11.1%) | 805 (12.1%) | 1039 (10.5%) | 0.001 |
| *Shock* | 719 (4.3%) | 276 (4.1%) | 443 (4.5%) | 0.351 |
| *Stroke* | 223 (0.7%) | 201 (0.7%) | 22 (0.9%) | 0.171 |
| *Torsades des point* | 229 (1.4%) | 59 (0.9%) | 170 (1.7%) | <0.001 |
| *Ventricular tachycardia* | 206 (1.2%) | 65 (1.0%) | 141 (1.4%) | 0.014 |
| Outcome |  |  |  |  |
| Admission life support limitation | 1570 (9.5%) | 566 (8.5%) | 1004 (10.1%) | <0.001 |
| ICU, intensive care unit  *Life support limitation includes any limitation including do not resuscitate (DNR), do not intubate (DNI), DNR-DNI, or comfort care only. | | | | |

**Appendix F**

Equation 1:

Logit(P(Y_Code Status_=1)) = β_0_ + β_1_•age_i_ + β_2_•sex_i_ + β_3_•race/ethnicity_i_ + β_4_•icu admission_i_ + β_5_•arrhythmia_i_ + β_6_•asthma_i_ + β_7_•blood loss anemia_i_ + β_8_•chronic kidney disease_i_ + β_9_•chronic pulmonary disease_i_ + β_10_•coagulopathy_i_ + β_11_•congestive heart failure_i_ + β_12_•coronary artery disease_i_ + β_13_•dementia_i_ + β_14_•diabetes mellitus_i_ + β_15_•esrd_i_ + β_16_•Hematologic malignancy_i_ + β_17_•HBV_i_ + β_18_•hcv_i_ + β_19_•hiv/aids or immunosuppression_i_ + β_20_•hld_i_ + β2_1_•htn_i_ + β_22_•hypothyroidism_i_ + β_23_•ida_i_ + β_24_•liver disease_i_ + β_25_•malnutrition_i_ + β_26_•metastatic cancer_i_ + β_27_•none_i_ + β_28_•obesity_i_ + β_29_•osa_i_ + β_30_•other_i_ + β_31_•paralysis_i_ + β_32_•pud_i_ + β_33_•psychosis_i_ + β_34_•pulmonary circulation disorder_i_ + β_35_•rheumatism_i_ + β_36_•transplant_i_ + β_37_•solid tumor without metastasis_i_ + β_38_•stroke_i_ + β_39_•sud_i_ + β_40_• vhd_i_ + β_41_•vte_i_ + β_42_•hypoxic respiratory failure_i_ + β_43_•acute liver injury_i_ + β_44_•acute myocardial infarction_i_ + β_45_•acute renal failure with dialysis_i_ + β_46_•Acute renal failure without dialysis_i_ + β_47_•ards_i_ + β_48_•afib_i_ + β_49_•cardiac arrest_i_ + β_50_•chf_i_ + β_51_•dka_i_ + β_52_•dic_i_ + β_53_•encephalopathy_i_ + β_54_•gib_i_ + β_55_•heart block_i_ + β_56_•hyperglycemia_i_ + β_57_•hypoglycemia_i_ + β_58_•meningoencephalitis_i_ + β_59_•myocarditis_i_ + β_60_•other_i_ + β_61_•pleural effusion_i_ + β_62_•pneumonia_i_ + β_63_•ptx_i_ + β_64_•rhabdo_i_ + β_65_•seizure_i_ + β_66_•sepsis_i_ + β_67_•shock_i_ + β_68_•stroke_i_ + β_69_•torsades_i_ + β_70_•vt_i_  + μ_j[i]_

| Table 4: Mixed-effect regression model odds ratio for exposure variable and all covariates for hospital variation. | | |
| --- | --- | --- |
|  | OR (95% CI) | *p*-value |
| Age (rescaled and centered, one standard deviation = 17.8 yrs) | 4.14 (3.89 – 4.42) | <0.001 |
| Sex (ref: male) | 1.32 (1.21 – 1.43) | <0.001 |
| Race/ethnicity (ref: non-Hispanic White) |  |  |
| *Asian* | 0.55 (0.41 – 0.75) | <0.001 |
| *Black / African American* | 0.52 (0.45 – 0.59) | <0.001 |
| *Hispanic* | 0.65 (0.56 – 0.75) | <0.001 |
| *Other* | 0.60 (0.45 – 0.80) | <0.001 |
| *Unknown* | 0.65 (0.43 – 0.99) | 0.045 |
| Comorbidities |  |  |
| *Arrhythmia* | 0.96 (0.84 – 1.11) | 0.604 |
| *Asthma* | 0.75 (0.63 – 0.89) | <0.001 |
| *Blood loss anemia* | 0.99 (0.72 – 1.35) | 0.933 |
| *Chronic kidney disease* | 1.18 (1.06 – 1.32) | 0.003 |
| *Chronic pulmonary disease* | 1.33 (1.20 -1.48) | <0.001 |
| *Coagulopathy* | 1.03 (0.85 – 1.26) | 0.752 |
| *Congestive heart failure* | 1.26 (1.12 – 1.43) | <0.001 |
| *Coronary artery disease* | 0.93 (0.83 – 1.05) | 0.256 |
| *Dementia* | 2.30 (2.04 – 2.60) | <0.001 |
| *Diabetes mellitus* | 0.99 (0.90 – 1.08) | 0.781 |
| *End-stage renal disease (ESRD)* | 1.14 (0.89 – 1.46) | 0.304 |
| *Hematologic malignancy* | 0.70 (0.52 – 0.95) | 0.024 |
| *Hepatitis B* | 1.94 (1.00 – 3.76) | 0.050 |
| *Hepatitis C* | 1.30 (0.93 – 1.81) | 0.122 |
| *HIV/AIDS or immunosuppression* | 1.77 (1.04 – 3.03) | 0.036 |
| *Hyperlipidemia* | 0.82 (0.73 – 0.92) | 0.001 |
| *Hypertension* | 0.92 (0.83 – 1.01) | 0.084 |
| *Hypothyroidism* | 1.12 (0.99 – 1.26) | 0.056 |
| *Iron deficiency anemia* | 1.12 (0.99 – 1.28) | 0.080 |
| *Liver disease* | 1.01 (0.81 – 1.26) | 0.915 |
| *Malnutrition* | 1.72 (1.32 – 2.26) | <0.001 |
| *Metastatic cancer* | 1.59 (1.26 – 1.99) | <0.001 |
| *None* | 0.75 (0.58 - 0.97) | 0.026 |
| *Obesity* | 0.76 (0.67 – 0.87) | <0.001 |
| *Obstructive sleep apnea* | 0.94 (0.78 – 1.12) | 0.473 |
| *Other* | 1.11 (0.98 – 1.24) | 0.090 |
| *Paralysis* | 2.35 (1.82 – 3.02) | <0.001 |
| *Peptic ulcer disease* | 1.14 (0.75 – 1.73) | 0.526 |
| *Psychosis* | 1.00 (0.74 – 1.35) | 0.992 |
| *Pulmonary circulation disorder* | 0.83 (0.58 – 1.19) | 0.301 |
| *Rheumatism* | 1.23 (0.95 – 1.59) | 0.113 |
| *Solid organ or bone marrow transplant* | 0.95 (0.55 – 1.64) | 0.850 |
| *Solid tumor without metastasis* | 1.30 (1.06 – 1.59) | 0.012 |
| *Stroke or other* | 1.36 (1.20 – 1.53) | <0.001 |
| *Substance use disorder* | 0.82 (0.63 – 1.06) | 0.128 |
| *Valvular heart disease* | 0.93 (0.78 – 1.11) | 0.449 |
| *Venous thromboembolism* | 1.04 (0.85 – 1.27) | 0.706 |
| Admission diagnosis |  |  |
| *Acute hypoxic respiratory failure* | 1.06 (0.98 – 1.24) | 0.256 |
| *Acute liver injury* | 0.86 (0.56 – 1.37) | 0.508 |
| *Acute myocardial infarction* | 1.35 (0.96 – 1.89) | 0.084 |
| *Acute renal failure with hemofiltration* | 1.51 (1.07 – 2.12) | 0.019 |
| *Acute renal failure without hemofiltration* | 1.00 (0.87 – 1.15) | 0.978 |
| *Acute respiratory distress syndrome* | 0.99 (0.77 – 1.26) | 0.925 |
| *Atrial fibrillation* | 0.94 (0.77 – 1.16) | 0.580 |
| *Cardiac arrest* | 1.03 (0.69 – 1.52) | 0.898 |
| *Congestive heart failure* | 1.19 (0.94 – 1.50) | 0.152 |
| *Diabetic ketoacidosis* | 1.00 (0.58 – 1.72) | 0.995 |
| *Disseminated intravascular coagulation* | 1.03 (0.50 – 2.17) | 0.922 |
| *Encephalopathy* | 1.39 (1.18 – 1.65) | <0.001 |
| *Gastrointestinal bleed* | 0.60 (0.40 – 0.91) | 0.015 |
| *Heart block* | 0.51 (0.31 – 0.82) | 0.006 |
| *Hyperglycemia* | 0.85 (0.66 – 1.11) | 0.244 |
| *Hypoglycemia* | 1.46 (1.00 – 2.12) | 0.048 |
| *Meningoencephalitis* | 1.60 (0.79 – 3.23) | 0.191 |
| *Myocarditis* | 0.71 (0.33 – 1.53) | 0.383 |
| *Other* | 1.00 (0.90 – 1.12) | 0.957 |
| *Pleural effusion* | 0.89 (0.62 – 1.26) | 0.501 |
| *Pneumonia* | 1.13 (0.94 – 1.34) | 0.188 |
| *Pneumothorax* | 0.66 (0.38 – 1.16) | 0.150 |
| *Rhabdomyolysis* | 1.63 (1.02 – 2.60) | 0.039 |
| *Seizure* | 0.75 (0.46 – 1.22) | 0.247 |
| *Sepsis* | 1.92 (1.69 – 2.19) | <0.001 |
| *Shock* | 0.69 (0.53 – 0.90) | 0.004 |
| *Stroke* | 0.76 (0.52 – 1.11) | 0.161 |
| *Torsades des Pointes* | 1.30 (0.76 – 2.23) | 0.343 |
| *Ventricular tachycardia* | 1.06 (0.71 – 1.57) | 0.789 |
| Other characteristics |  |  |
| ICU admission | 0.97 (0.88 – 1.08) | 0.598 |

**Appendix G**

Equation 2:

Logit(P(Y_Code Status_=1)) = β_0_ + β_1_•age_i_ + β_2_•sex_i_ + β_3_•race/ethnicity_i_ + β_4_•icu admission_i_ + β_5_•arrhythmia_i_ + β_6_•asthma_i_ + β_7_•blood loss anemia_i_ + β_8_•chronic kidney disease_i_ + β_9_•chronic pulmonary disease_i_ + β_10_•coagulopathy_i_ + β_11_•congestive heart failure_i_ + β_12_•coronary artery disease_i_ + β_13_•dementia_i_ + β_14_•diabetes mellitus_i_ + β_15_•esrd_i_ + β_16_•Hematologic malignancy_i_ + β_17_•HBV_i_ + β_18_•hcv_i_ + β_19_•hiv/aids or immunosuppression_i_ + β_20_•hld_i_ + β2_1_•htn_i_ + β_22_•hypothyroidism_i_ + β_23_•ida_i_ + β_24_•liver disease_i_ + β_25_•malnutrition_i_ + β_26_•metastatic cancer_i_ + β_27_•none_i_ + β_28_•obesity_i_ + β_29_•osa_i_ + β_30_•other_i_ + β_31_•paralysis_i_ + β_32_•pud_i_ + β_33_•psychosis_i_ + β_34_•pulmonary circulation disorder_i_ + β_35_•rheumatism_i_ + β_36_•transplant_i_ + β_37_•solid tumor without metastasis_i_ + β_38_•stroke_i_ + β_39_•sud_i_ + β_40_• vhd_i_ + β_41_•vte_i_ + β_42_•hypoxic respiratory failure_i_ + β_43_•acute liver injury_i_ + β_44_•acute myocardial infarction_i_ + β_45_•acute renal failure with dialysis_i_ + β_46_•Acute renal failure without dialysis_i_ + β_47_•ards_i_ + β_48_•afib_i_ + β_49_•cardiac arrest_i_ + β_50_•chf_i_ + β_51_•dka_i_ + β_52_•dic_i_ + β_53_•encephalopathy_i_ + β_54_•gib_i_ + β_55_•heart block_i_ + β_56_•hyperglycemia_i_ + β_57_•hypoglycemia_i_ + β_58_•meningoencephalitis_i_ + β_59_•myocarditis_i_ + β_60_•other_i_ + β_61_•pleural effusion_i_ + β_62_•pneumonia_i_ + β_63_•ptx_i_ + β_64_•rhabdo_i_ + β_65_•seizure_i_ + β_66_•sepsis_i_ + β_67_•shock_i_ + β_68_•stroke_i_ + β_69_•torsades_i_ + β_70_•vt_i_  + β_71_•pandemic era + μ_j[i]_

| Table 5: Mixed-effect regression model odds ratio for exposure variable and all covariates for temporal variation. | | |
| --- | --- | --- |
|  | OR (95% CI) | *p*-value |
| Age (rescaled, one standard deviation) | 3.95 (3.61 – 4.34) | <0.001 |
| Sex (ref: male) | 1.25 (1.11 – 1.41) | <0.001 |
| Race/ethnicity (ref: non-Hispanic White) |  |  |
| *Asian* | 0.67 (0.44 – 1.03) | 0.065 |
| *Black / African American* | 0.51 (0.41 – 0.63) | <0.001 |
| *Hispanic* | 0.59 (0.45 – 0.76) | <0.001 |
| *Other* | 0.83 (0.56 – 1.24) | 0.370 |
| *Unknown* | 0.61 (0.39 – 0.97) | 0.035 |
| Comorbidities |  |  |
| *Arrhythmia* | 0.85 (0.68 – 1.06) | 0.154 |
| *Asthma* | 0.75 (0.63 – 0.89) | <0.001 |
| *Blood loss anemia* | 0.72 (0.37 – 1.40) | 0.332 |
| *Chronic kidney disease* | 1.16 (0.99 – 1.36) | 0.062 |
| *Chronic pulmonary disease* | 1.35 (1.15 -1.59) | <0.001 |
| *Coagulopathy* | 1.06 (0.74 – 1.51) | 0.756 |
| *Congestive heart failure* | 1.34 (1.10 – 1.64) | 0.003 |
| *Coronary artery disease* | 0.93 (0.79 – 1.09) | 0.358 |
| *Dementia* | 2.61 (2.12 – 3.20) | <0.001 |
| *Diabetes mellitus* | 0.98 (0.86 – 1.13) | 0.823 |
| *End-stage renal disease* | 1.14 (0.76 – 1.71) | 0.511 |
| *Hematologic malignancy* | 0.62 (0.39 – 0.97) | 0.035 |
| *Hepatitis B* | 2.31 (0.91 – 5.92) | 0.079 |
| *Hepatitis C* | 1.42 (0.76 – 2.64) | 0.266 |
| *HIV/AIDS or immunosuppression* | 1.86 (0.94 – 3.70) | 0.075 |
| *Hyperlipidemia* | 0.92 (0.81 – 1.07) | 0.289 |
| *Hypertension* | 1.01 (0.88 – 1.17) | 0.836 |
| *Hypothyroidism* | 1.26 (1.03 – 1.55) | 0.024 |
| *Iron deficiency anemia* | 1.15 (0.88 – 1.49) | 0.308 |
| *Liver disease* | 1.09 (0.72 – 1.64) | 0.686 |
| *Malnutrition* | 2.13 (1.28 – 3.52) | 0.003 |
| *Metastatic cancer* | 1.17 (0.86 – 1.59) | 0.319 |
| *None* | 0.73 (0.48 - 1.11) | 0.139 |
| *Obesity* | 0.64 (0.52 – 0.80) | <0.001 |
| *Obstructive sleep apnea* | 0.91 (0.72 – 1.15) | 0.419 |
| *Other* | 1.10 (0.96 – 1.27) | 0.176 |
| *Paralysis* | 2.39 (1.10 – 5.21) | 0.028 |
| *Peptic ulcer disease* | 0.94 (0.46 – 1.93) | 0.865 |
| *Psychosis* | 1.13 (0.74 – 1.72) | 0.573 |
| *Pulmonary circulation disorder* | 1.20 (0.58 – 2.48) | 0.623 |
| *Rheumatism* | 1.04 (0.69 – 1.55) | 0.858 |
| *Solid organ or bone marrow transplant* | 1.09 (0.55 – 2.18) | 0.805 |
| *Solid tumor without metastasis* | 1.31 (0.94 – 1.84) | 0.111 |
| *Stroke or other* | 1.14 (0.91 – 1.43) | 0.246 |
| *Substance use disorder* | 0.87 (0.54 – 1.36) | 0.510 |
| *Valvular heart disease* | 1.01 (0.73 – 1.40) | 0.933 |
| *Venous thromboembolism* | 0.83 (0.61 – 1.13) | 0.236 |
| Admission diagnosis |  |  |
| *Acute hypoxic respiratory failure* | 1.01 (0.88 – 1.16) | 0.877 |
| *Acute liver injury* | 1.31 (0.63 – 2.73) | 0.470 |
| *Acute myocardial infarction* | 1.20 (0.70 – 2.06) | 0.496 |
| *Acute renal failure with hemofiltration* | 1.68 (1.05 – 2.70) | 0.031 |
| *Acute renal failure without hemofiltration* | 1.02 (0.85 – 1.22) | 0.863 |
| *Acute respiratory distress syndrome* | 1.02 (0.71 – 1.48) | 0.899 |
| *Atrial fibrillation* | 1.10 (0.85 – 1.43) | 0.475 |
| *Cardiac arrest* | 0.80 (0.41 – 1.57) | 0.521 |
| *Congestive heart failure* | 1.17 (0.84 – 1.63) | 0.353 |
| *Diabetic ketoacidosis* | 0.92 (0.42 – 2.02) | 0.831 |
| *Disseminated intravascular coagulation* | 0.82 (0.23 – 2.98) | 0.769 |
| *Encephalopathy* | 1.57 (1.24 – 1.98) | <0.001 |
| *Gastrointestinal bleed* | 0.54 (0.29 – 1.00) | 0.050 |
| *Heart block* | 0.39 (0.16 – 0.96) | 0.041 |
| *Hyperglycemia* | 0.82 (0.58 – 1.15) | 0.252 |
| *Hypoglycemia* | 1.38 (0.78 – 2.43) | 0.272 |
| *Meningoencephalitis* | 1.13 (0.37 – 3.47) | 0.834 |
| *Myocarditis* | 0.31 (0.07 – 1.29) | 0.107 |
| *Other* | 0.94 (0.80 – 1.10) | 0.438 |
| *Pleural effusion* | 0.82 (0.50 – 1.34) | 0.429 |
| *Pneumonia* | 1.21 (0.97 – 1.52) | 0.922 |
| *Pneumothorax* | 0.69 (0.30 – 1.58) | 0.377 |
| *Rhabdomyolysis* | 1.28 (0.64 – 2.59) | 0.487 |
| *Seizure* | 0.82 (0.40 – 1.67) | 0.582 |
| *Sepsis* | 1.59 (1.32 – 1.93) | <0.001 |
| *Shock* | 0.89 (0.61 – 1.30) | 0.550 |
| *Stroke* | 0.69 (0.38 – 1.29) | 0.246 |
| *Torsades des Pointes* | 1.68 (0.75 – 3.77) | 0.207 |
| *Ventricular tachycardia* | 1.31 (0.75 – 2.30) | 0.347 |
| Other characteristics |  |  |
| ICU admission | 1.05 (0.92 – 1.22) | 0.457 |
| Admission timeline |  |  |
| Late pandemic (ref: early pandemic) | 0.92 (0.79 – 1.07) | 0.257 |

**Appendix H**

| Table 6: Mixed-effect regression model results for both hospital and temporal variation. | | |
| --- | --- | --- |
|  | OR (95% CI) | *p*-value |
| *Model 1* (n = 42,383 with 75 hospitals; 31,677 with 74 hospitals) | | |
| Hospital of admission (complete cases, imputed) | 1.85 (1.60 – 2.14) *^†^* | - |
| Hospital of admission (complete cases, non-imputed) *^††^* | 1.81 (1.60 – 2.14) *^†^* | - |
| *Model 2* (n = 16,801) | | |
| Admission during late pandemic (ref = early pandemic) | 0.92 (0.79 – 1.07) | 0.267 |
| *^†^*Refers to the median odds ratio.  *^††^*Sensitivity analysis using complete cases from entire cohort without imputation.  Additional covariate results are reported in the Supplementary Material | | |

**Appendix I**

**Figure 2:** All covariates odds ratio for likelihood of having life support limitation on admission (n = 42,383).

**
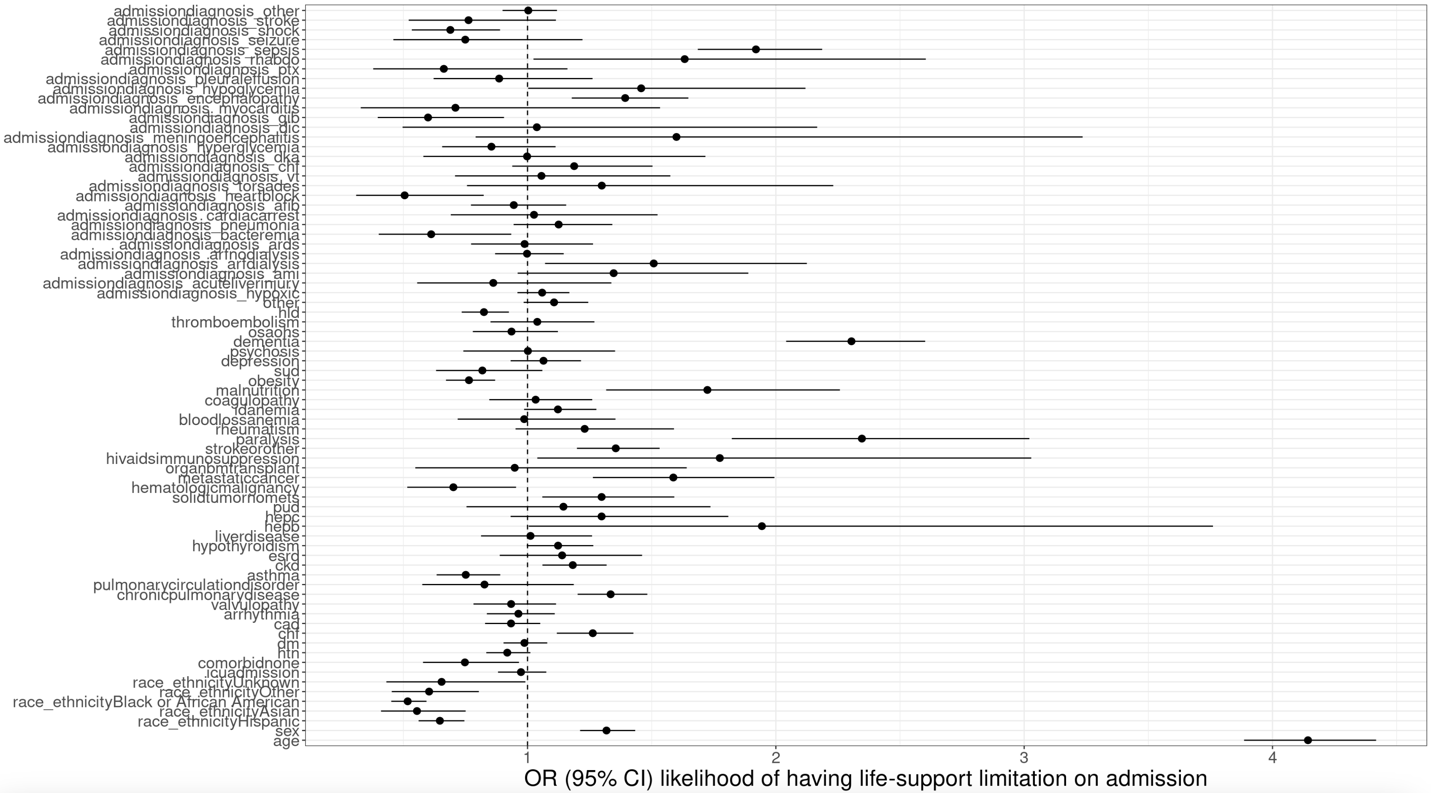
**

**Figure 3:** The unadjusted and adjusted monthly rate of life-support limitation amongst cohort with admission information available (n = 16,581)

**
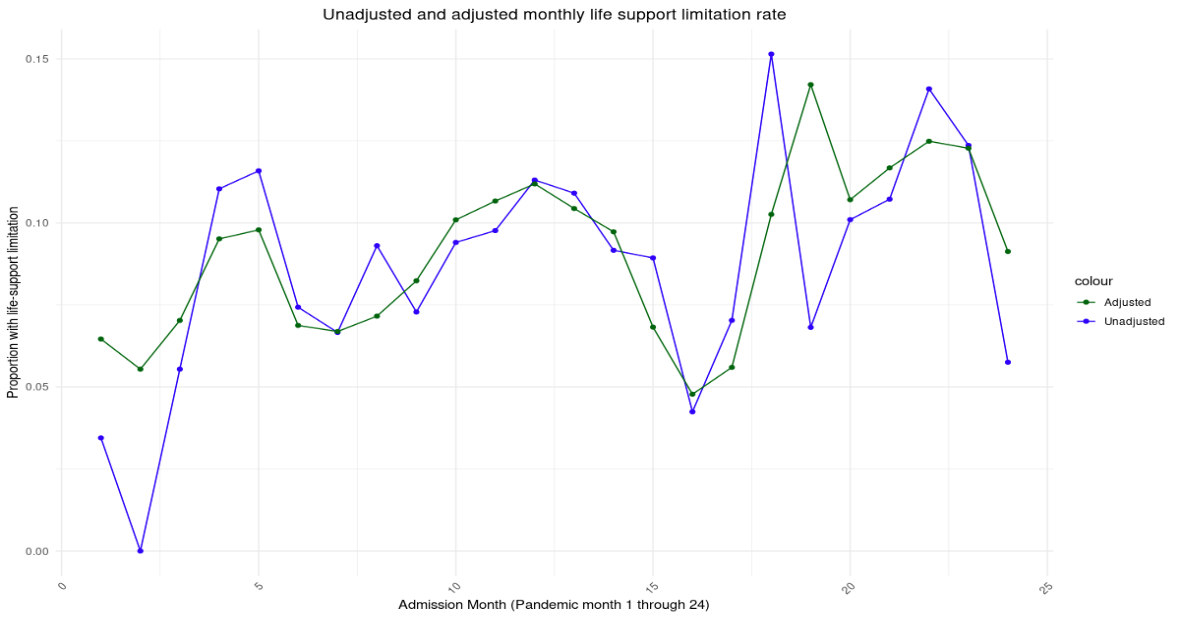
**

**Appendix J**

**Figure 4:** Receiver operating curve for predicting life support limitation upon admission based on only baseline demographic, then with added comorbidities and admission diagnoses, and then with addition of hospital of admission.


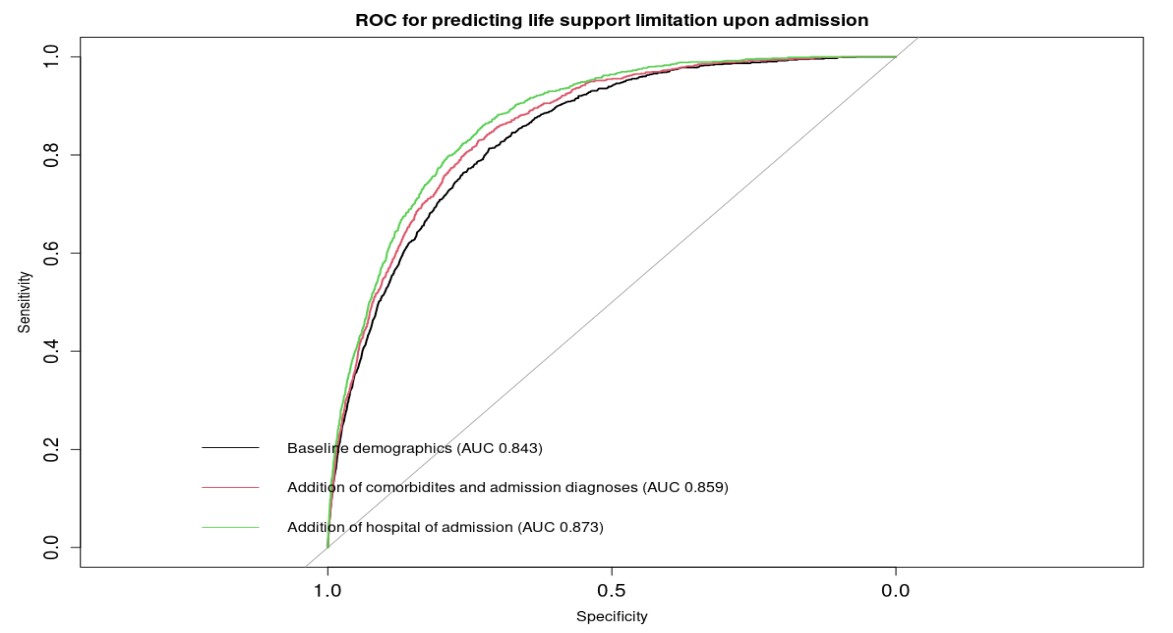
Model_baseline_: AIC = 18209.

Model_baseline + comorbidities and diagnoses_: AIC = 17546.

Model_baseline, comorbidities, diagnosis + hospital_: AIC = 17043.

*Baseline:*

Logit(P(Y_Code Status_=1)) = β_0_ + β_1_•age_i_ + β_2_•sex_i_ + β_3_•race/ethnicity_i_ + β_4_•icu admission_i_

*Baseline, comorbidities, and diagnoses:*

Logit(P(Y_Code Status_=1)) = β_0_ + β_1_•age_i_ + β_2_•sex_i_ + β_3_•race/ethnicity_i_ + β_4_•icu admission_i_ + β_5_•arrhythmia_i_ + β_6_•asthma_i_ + β_7_•blood loss anemia_i_ + β_8_•chronic kidney disease_i_ + β_9_•chronic pulmonary disease_i_ + β_10_•coagulopathy_i_ + β_11_•congestive heart failure_i_ + β_12_•coronary artery disease_i_ + β_13_•dementia_i_ + β_14_•diabetes mellitus_i_ + β_15_•esrd_i_ + β_16_•Hematologic malignancy_i_ + β_17_•HBV_i_ + β_18_•hcv_i_ + β_19_•hiv/aids or immunosuppression_i_ + β_20_•hld_i_ + β2_1_•htn_i_ + β_22_•hypothyroidism_i_ + β_23_•ida_i_ + β_24_•liver disease_i_ + β_25_•malnutrition_i_ + β_26_•metastatic cancer_i_ + β_27_•none_i_ + β_28_•obesity_i_ + β_29_•osa_i_ + β_30_•other_i_ + β_31_•paralysis_i_ + β_32_•pud_i_ + β_33_•psychosis_i_ + β_34_•pulmonary circulation disorder_i_ + β_35_•rheumatism_i_ + β_36_•transplant_i_ + β_37_•solid tumor without metastasis_i_ + β_38_•stroke_i_ + β_39_•sud_i_ + β_40_• vhd_i_ + β_41_•vte_i_ + β_42_•hypoxic respiratory failure_i_ + β_43_•acute liver injury_i_ + β_44_•acute myocardial infarction_i_ + β_45_•acute renal failure with dialysis_i_ + β_46_•Acute renal failure without dialysis_i_ + β_47_•ards_i_ + β_48_•afib_i_ + β_49_•cardiac arrest_i_ + β_50_•chf_i_ + β_51_•dka_i_ + β_52_•dic_i_ + β_53_•encephalopathy_i_ + β_54_•gib_i_ + β_55_•heart block_i_ + β_56_•hyperglycemia_i_ + β_57_•hypoglycemia_i_ + β_58_•meningoencephalitis_i_ + β_59_•myocarditis_i_ + β_60_•other_i_ + β_61_•pleural effusion_i_ + β_62_•pneumonia_i_ + β_63_•ptx_i_ + β_64_•rhabdo_i_ + β_65_•seizure_i_ + β_66_•sepsis_i_ + β_67_•shock_i_ + β_68_•stroke_i_ + β_69_•torsades_i_ + β_70_•vt_i_

**Figure 5:** Calibration plot for final model which includes baseline characteristics, comorbidities, diagnoses, and hospital of admission.


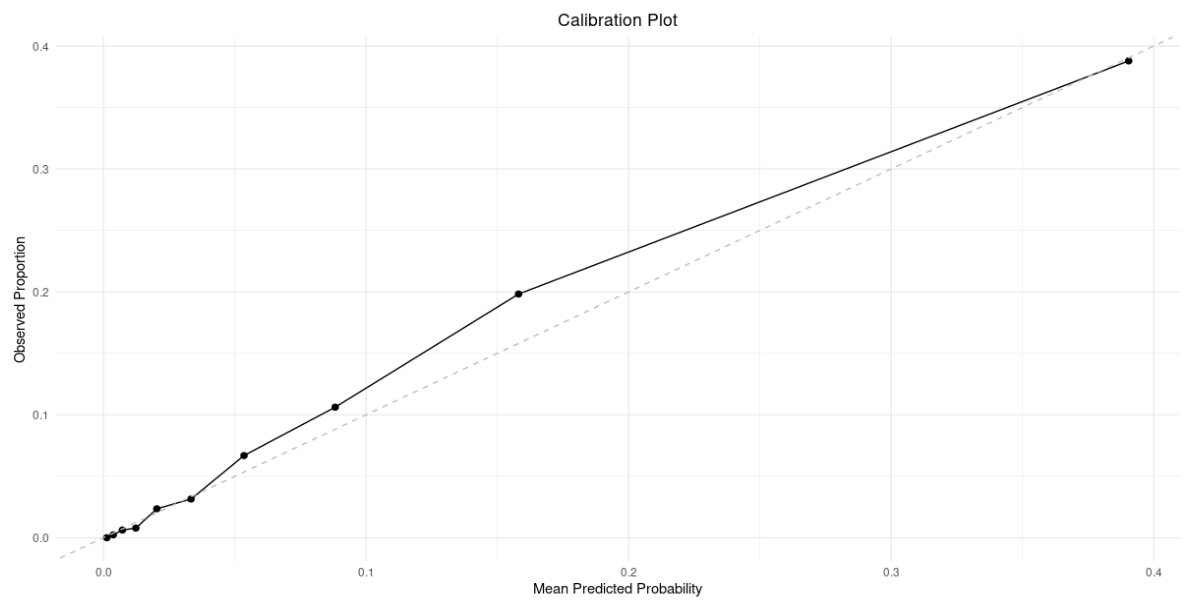


**Appendix K**

**Collaborative contributors to the VIRUS REGISTRY from relevant sites.**

**United States**

Baptist Health South Florida: Donna Lee Armaignac, Don Parris, Maria Pilar Zuniga, Ilea Vargas, Viviana Boronat, Anneka Hutton, Navneet Kaur, Prashank Neupane, Nohemi Sadule-Rios, Lourdes M. Rojas, Aashish Neupane, Priscilla Rivera, Carlos Valle Carlos, Gregory Vincent

Greenville Memorial Hospital: Nicholas Perkins, Prerana Roth, Alain Litwin, Anand Pariyadath, Phillip Moschella, Trayson Llano, Lior Rennert

Thomas Jefferson University Hospital: Katherine A. Belden, Michael Baram, Devin M. Weber, Rosalie DePaola, Yuwei Xia, Hudson Carter, Aaron Tolley, Mary Ferranti

Mayo Clinic Rochester: Rahul Kashyap, Juan Pablo Domecq, Ognjen Gajic, Vikas Bansal, Aysun Tekin, Amos Lal, John C. O'Horo, Neha N. Deo, Mayank Sharma, Shahraz Qamar, Romil Singh, Diana J. Valencia Morales

Allina Health (Abbott Northwestern Hospital, United Hospital and Mercy Hospital in Minnesota): Roman R. Melamed, Joan Hall, David Tierney, Love Patel, Lisa Kirkland, Ramiro Saavedra-Romero, Justin Kirven, Ben Krehbiel, Nova Schmitz, Cecely Hoyt, Anna Schulte, Abbey Sidebottom, and Whitney Wunderlic, and Ngoc Ha

Wake Forest University School of Medicine; Wake Forest Baptist Health Network: Ashish K. Khanna, Lynnette Harris, Bruce Cusson, Brandon Reeves, Jessica Fanelli, Nataya Disher, Anusha Samant, Chritian DeGroot, Evan Youshock, R. Miller Ligon, Katherine McCartney, Julio Garcia, Chidi Iloabachie, Kelsey Flores, David VanEenenaam, Lauren Sands, Samuel Robinson, Nia Sweatt, Jacob Fowler, Madeline Fram, Easton Howard, Kathleen Johnson, Imoh Udoh, Lillian Nosow, Lucy Winesett Howard, Quan Duc Minh Pham, Aman Irfanullah, Tiffany Ong, Chukwunyelu Henry Enwezor, Hannah Dabagian, Rafael Mendoza

Medical College of Wisconsin: Rahul S Nanchal, Paul A Bergl, Jennifer L Peterson

Beth Israel Deaconess Medical Center: Valerie Banner Goodspeed, Lauren Kelly, Krystal Capers, Melisa Joseph, Lauryn Tsai.

Atrium Health Navicent: Amy B. Christie, Dennis W. Ashley, Rajani Adiga

Mayo Clinic Arizona: Rodrigo Cartin-Ceba, Ayan Sen, Fahimeh Talaei

Mayo Clinic, Eau Claire: Abigail T. La Nou, Marija Bogojevic, Simon Zec

Mayo Clinic, Florida: Devang Sanghavi, Pramod Guru, Pablo Moreno Franco, Karthik Gnanapandithan, Hollie Saunders, Zachary Fleissner, Juan Garcia , Alejandra Yu Lee Mateus, Siva Naga Yarrarapu, Nirmaljot Kaur, Abhisekh Giri, Mohammed Mustafa Hasan, Ashrita Donepudi

Valleywise Health (formerly Maricopa Medical Center): Murtaza Akhter, Rania Abdul Rahman, Mary Mulrow

Mayo Clinic Rochester: Rahul Kashyap, Juan Pablo Domecq, Ognjen Gajic, Vikas Bansal, Aysun Tekin, Amos Lal, John C. O'Horo, Neha N. Deo, Mayank Sharma, Shahraz Qamar, Romil Singh, Diana J. Valencia Morales

Clements University Hospital at UT Southwestern Medical Center: Sreekanth Cheruku, Farzin Ahmed, Christopher Deonarine, Ashley Jones, Mohammad-Ali Shaikh, David Preston, Jeanette Chin

St. Joseph Mercy Ann Arbor, Ann Arbor: Harry L. Anderson, III, Dixy Rajkumar, Ali Abunayla, Jerrilyn Heiter

Tulane University Medical Center and University Medical Center New Orleans: Joshua L. Denson, A. Scott Gillet, Margo Brown, Rachael Stevens, Andrew Wetherbie, Kevin Tea, Mathew Moore

MetroHealth Medical Center: Yasir Tarabichi, Adam Perzynski, Christine Wang, Dhatri Kotekal

Boston University School of Medicine, Boston, MA: Allan J. Walkey, Sushrut S. Waikar, Michael A. Garcia, Mia Colona, Zoe Kibbelaar, Michael Leong, Daniel Wallman, Kanupriya Soni, Jennifer Maccarone, Joshua Gilman, Ycar Devis, Joseph Chung, Munizay Paracha, David N. Lumelsky, Madeline DiLorenzo, Najla Abdurrahman, Shelsey Johnson

University of Cincinnati: Dina Gomaa B.S., Michael Goodman, Devin Wakefield, Anthony Spuzzillo, John O. Shinn II

Banner University Medical Center-Tucson: Jarrod M Mosier, Karen Lutrick, Beth Salvagio Campbell, Cathleen Wilson, Patrick Rivers, Jonathan Brinks, Mokenge Ndiva Mongoh, Boris Gilson

St.Agnes Hospital: Anthony Martinez, Micheal Allison, Aniket Mittal, Rafael Ruiz, Aleta Skaanland, Robert Ross

Howard University Hospital: Norma Smalls

Chambersburg Hospital: Raghavendra Tirupathi, Alymer Tang, Arshad Safi, Cindy Green, Jackie Newell, Naga Ramani, Bhavani Harika Ganti

UC San Diego Medical Center – Hillcrest: Abdurrahman Husain, Atul Malhotra, Qais Zawaydeh

Advocate Christ Medical Center: Kenneth W. Dodd, Nicholas Goodmanson, Kathleen Hesse,Paige Bird, Chauncey Weinert, Nathan Schoenrade, Abdulrahman Altaher, Esmael Mayar, Matthew Aronson, Tyler Cooper, Monica Logan, Brianna Miner, Gisele Papo

The University of Tennessee Medical Center: Caleb Darby, Kristy Page, Amanda Brown, Jessie McAbee

Cox Medical Center Springfield: Steven K. Daugherty, Sam Atkinson, Kelly Shrimpton

Buffalo General Medical Center: Kimberly Zammit, Patrick, J, McGrath, William, Loeffler,Maya, R, Chilbert

Mercy Hospital, Saint Louis: Chakradhar Venkata, Miriam Engemann, Annamarie Mantese

Texas Health Resources, Arlington: Kristina L Carter, PharmD, Michael A Olmos, Brittany M Parker, Julio Quintanilla, Tara A Craig, Brendon J Clough, Jeffrey T Jameson

St. Joseph's Candler Health System: Howard A. Zaren, Stephanie J. Smith, Grant C. Lewis, Lauren Seames, Cheryl Farlow, Judy Miller, Gloria Broadstreet

Christus Spohn Shoreline Corpus Christi: Salim Surani, Joshua White, Sunny Italiya, and Rahul Dhadhwal

Brooke Army Medical Center: Maj Andrew M. Hersh, CPT Stephanie L Wachs, Brittany S. Swigger, CPT Stephanie L Wachs, Capt Lauren A. Sattler, Capt Michael N. Moulton

Hospital HIMA San Pablo Caguas: Gloria Rodriguez, Zoraida DelValle, Wilfredo DeJesus, Darleen Gonzalez, Migdalia Arce

Cleveland Clinic (Main Campus, Fairview Hospital, Florida-Weston, Hillcrest Hospital, Marymount Hospital): Vidula Vachharajani, Abhijit Duggal, Prabalini Rajendram, Omar Mehkri, Siddharth Dugar, Michelle Biehl, Gretchen Sacha, , Stuart Houltham, Alexander King, Kiran Ashok, Bryan Poynter, Mary Beukemann, Richard Rice, Susan Gole, Valerie Shaner, Adarsh Conjeevaram, Michelle Ferrari, Narendrakumar Alappan, Steven Minear, Jaime Hernandez-Montfort, Syed Sohaib Nasim, Ravi Sunderkrishnan, Debasis Sahoo

Stamford Health: Michael A. Bernstein, Ian K. Goff, Matthew Naftilan, Amal Mathew, Deborah Williams, Sue Murdock, RN, Maryanne Ducey, Kerianne Nelson

The Children's Hospital at OU Medicine: Neha Gupta, Tracy L Jones, Shonda C Ayers, Amy B Harrell, Brent R Brown, OU Medical Center: Neha Gupta, Brent R Brown, Tracy L Jones, Kassidy Malone, Lauren A Sinko, Amy B Harrell, Shonda C Ayers, Lisa M Settle, Taylor J Sears

Baylor Scott & White Health: Valerie C. Danesh, Gueorgui Dubrocq, Amber L. Davis, Marissa J Hammers, ill M. McGahey, Amanda C. Farris, Elisa Priest, Robyn Korsmo, Lorie Fares, Kathy Skiles, Susan M. Shor, Kenya Burns, Corrie A Dowell, Gabriela “Hope” Gonzales, Melody Flores, Lindsay Newman, Debora A Wilk, Jason Ettlinger, Jaccallene Bomar, Himani Darji, Alejandro Arroliga, Alejandro C Arroliga, Corrie A. Dowell, Gabriela Hope Conzales, Melody Flores, Lindsay Newman, Debora A. Wilk, Jason Ettlinger, Himani Darji, Jaccallene Bomar

Lakes Region General Hospital: Michael Smith, William Snow, Riley Liptak, Hannah Durant, Valerie Pendleton, Alay Nanavati, Risa Mrozowsk, Erica Doubleday

OSF Saint Francis Medical Center: Bhagat S. Aulakh, Sandeep Tripathi, Jennifer A. Bandy, Lisa M. Kreps, Dawn R. Bollinger

Parkview Health System, Fort Wayne: Roger Scott Stienecker, Andre G. Melendez, Tressa A. Brunner, Sue M Budzon, Jessica L. Heffernan, Janelle M. Souder, Tracy L. Miller, Andrea G. Maisonneuve

Ascension/St. Thomas Research Institute West Campus: Stephen Capizzi, Bethany Alicie, Martha Green, Lori Crockarell, Amelia Drennan, Kathleen Dubuque, Tonya Fambrough, Nikole Gasaway, Briana Krantz, Peiman Nebi, Jan Orga, Margaret Serfass, Alina Simion, Kimberly Warren, Cassie Wheeler, CJ Woolman

MacNeal Hospital Loyola Medicine: Christine C. Junia, Robert Lichtenberg, Hasrat Sidhu, Diana Espinoza, Shelden Rodrigues, Maria Jose Zabala, Daniela Goyes, Ammu Susheela, Buddhi Hatharaliyadda, Naveen Rameshkumar, Amulya Kasireddy, Genessis Maldonado, Lisseth Beltran, Akshata Chaugule, Hassan Khan

Duke University Hospital: Raquel R Bartz, Vijay Krishnamoorthy, Bryan Kraft, Aaron Pulsipher, Eugene Friedman, Sachin Mehta

Ridgecrest Regional Hospital: Victoria Schauf, Chris Wall,

University of Michigan Health System: Pauline Park, Andrew Admon, Sinan Hanna, Rishi Chanderraj, Maria Pliakas, Ann Wolski, Jennifer Cirino

University of Vermont Larner College of Medicine: Renee D. Stapleton, Anne E. Dixon, Olivia Johnson, Sara S. Ardren, Stephanie Burns, Anna Raymond, Erika Gonyaw, Kevin Hodgdon, Chloe Housenger, Benjamin Lin, Karen McQuesten, Heidi Pecott-Grimm, Julie Sweet, Sebastian Ventrone

Baylor College of Medicine, Baylor St. Lukes Medical Center: Christopher M Howard, Cameron McBride, Jocelyn Abraham, Orlando Garner, Katherine Richards, Keegan Collins, Preethi Antony, Sindhu Mathew

JPS Health Network: Steven Q. Davis, Valentina Jovic, Valentina Jovic, Max Masuda, Amanda Hayes

SUNY Upstate Medical University: Ioana Amzuta, Amish Shah, Ritu Modi, Hassan Al-Khalisy, Pardeep Masuta, Melissa Schafer, Angela Wratney

George Washington University: David P. Yamane, Ivy Benjenk, Nivedita Prasanna

Detar Family Medicine residency: Sidney Ontai, Brian Contreras, MD, Uzoma Obinwanko, Nneka Amamasi, Amir Sharafi

Sarasota Memorial Hospital: Antonia L. Vilella, Sara B. Kutner, Kacie Clark, Danielle Moore

University of Kansas Medical Center: Usman Nazir, Garrett Rampon, Jake Riggle, Nathan Dismang

University of Washington – Northwest: Nita Khandelwal, T. Eoin West, Ellen S. Caldwell, Lara Lovelace-Macon, Navya Garimella, Denisse B. Dow

Advocate/Aurora St Luke's Medical Center: Eric M. Siegal, Phyllis Runningen

University of Miami Miller School of Medicine: Roger A. Alvarez, Amarilys Alarcon-Calderon, Marie Anne Sosa, Sunita K. Mahabir, Mausam J. Patel

University of Pittsburgh: Faraaz Ali Shah, Byron Chuan, Sagar L. Rawal, Manal Piracha

University of Utah Health: Joseph E. Tonna, Nicholas M. Levin, Kayte Suslavich, Rachel Tsolinas, Zachary T. Fica, Chloe R. Skidmore

University Hospital San Antonio: Emily A. Vail, Susannah Nicholson, Rachelle B. Jonas, AnnaRose E. Dement, William Tang, Mark DeRosa, Robert E. Villarreal

Saint Alphonsus Regional Medical Center: Kerry P. J. Pulver, Jennifer Yehle, Alicia Weeks, Terra Inman

ProMedica Toledo Hospital: Roberta E. Redfern, Jessica Shoemaker, Jennifer Micham, Lynn Kenney, Gabriel Naimy

St.Mary Medical Center, Langhorne: Umang Patel, Jordesha Hodge, KrunalKumar Patel, Shivani Dalal, Himanshu Kavani, Sam Joseph

University Medical Center (University Medical Center of Southern Nevada Las Vegas): Rajany V. Dy, Alfredo Iardino, Jill Sharma, Richard Czieki, Julia Christopher, Ryan Lacey, Marwan Mashina,, Kushal Patel

Samaritan Health Services: Brian L. Delmonaco, Anthony Franklin, Mitchell Heath

University of Florida Health Shands Hospital: Azra Bihorac, Tezcan Ozrazgat Baslanti, George Omalay, Haleh Hashemighouchani, Julie S. Cupka, Matthew M Ruppert

Augusta Health: Andrew S. Moyer, George M. Verghese

Gundersen Health System La Crosse Wisconsin: Christine Waller, Kara Kallies, Christine Waller, Jonean Thorsen, Alec Fitzsimmons, Haley Olsen

Millard Fillmore Suburban Hospital: Anna Eschler, Mary Hejna, Emily Lewandowski, Kristen Kusmierski, Clare Martin

University of Iowa Carver College of Medicine: Patrick W. McGonagill, Colette Galet, Janice Hubbard, David Wang, Lauren Allan, Aditya Badheka, Madhuradhar Chegondi

Washington University School of Medicine and Barnes-Jewish Hospital: Patrick G. Lyons, Andrew P Michelson, Sara S. Haluf, Lauren M. Lynch, Nguyet M. Nguyen, Aaron Steinberg

AnMed Health: Abhijit A Raval, Andrea Franks

M Health-Fairview, University of Minnesota: Ronald A. Reilkoff, Julia A. Heneghan, Sarah Eichen, Lexie Goertzen, Scott Rajala, Ghislaine Feussom, Ben Tang

Wyoming Medical Center: Vishwanath Pattan, Jessica Papke, Ismail Jimada, Nida Mhid, Samuel Chakola
